# Supplementary material for: Comparative analysis of COVID-19 vaccine responses and third booster dose-induced neutralizing antibodies against Delta and Omicron variants
Source: Nat Commun. 2022 May 5;13:2476. doi: 10.1038/s41467-022-30162-5 (PMC9072399; doi:10.1038/s41467-022-30162-5)
Supplement: Supplementary file 1 — Supplementary information [file 41467_2022_30162_MOESM1_ESM.pdf]

Comparative analysis of COVID-19 vaccine responses and third booster dose-induced neutralizing antibodies against Delta and Omicron variants

By Belik et. al

Supplementary Figures 1-4

Supplementary Table 1

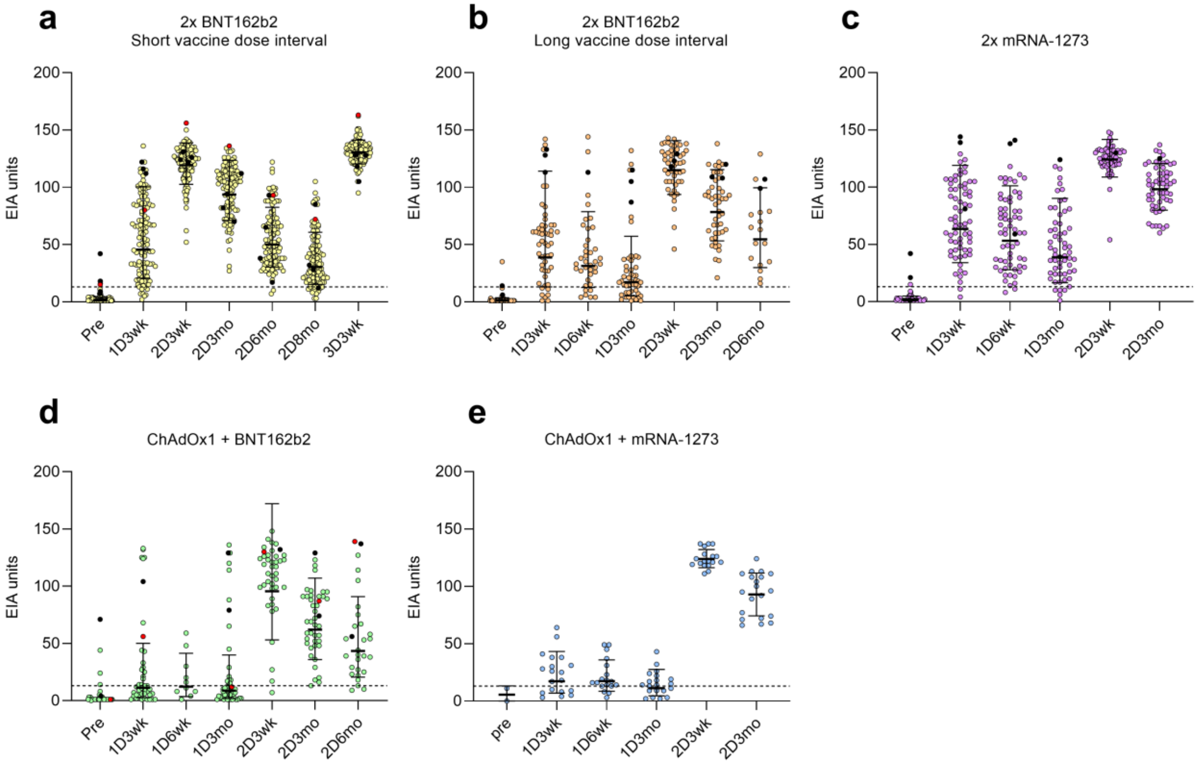

Supplementary Figure 1. SARS-CoV-2 S1-specific antibody responses of HCWs with a serum dilution of 1:300. SARS-CoV-2 S1-specific IgG antibody levels were measured in all vaccine combination groups (2x BNT162b2 (short and long dose interval, **a** and **b**), 2x mRNA-1273 (**c**), ChAdOx1 + BNT162b2 (**d**), and ChAdOx1 + mRNA-1273 (**e**)) with EIA using a 1:300 serum dilution. Serum samples from HCWs in the short dose interval group were collected before the vaccination (Pre), three weeks after the first dose (1D3wk), three weeks (2D3wk), three months (2D3mo), six months (2D6mo), and eight months (2D8mo) after the second vaccine dose (2D). Serum samples from HCWs in the long dose interval group were collected before vaccination (Pre), three weeks (1D3wk), six weeks (1D6wk) and twelve weeks (1D12wk) after the first vaccine dose (1D), and three weeks (2D3wk), three months (2D3mo), and six months (2D6mo), after the second vaccine dose (2D). Sample amounts are represented in Supplementary Table 1. Twelve HCWs with PCR confirmed SARS-CoV-2 infection prior to vaccinations are represented with black dots and two HCWs with breakthrough infections (>20 EIA unit increase between samples or positive PCR test result) are represented with red dots. Geometric mean antibody levels are shown as lines with geometric SD. Cut-off value is indicated with a dashed line.

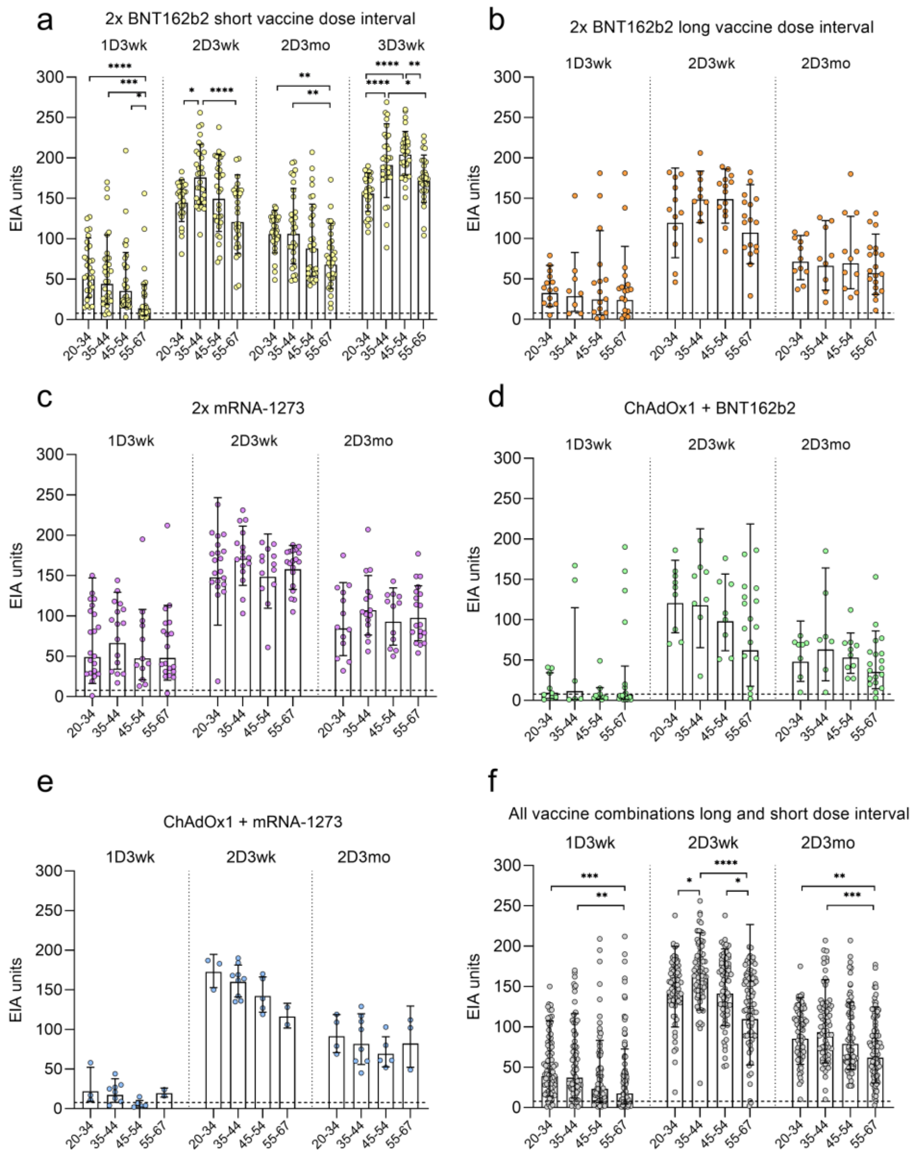

Supplementary Figure 2. SARS-CoV-2 S1-specific antibody responses of HCWs after one, two, or three COVID-19 vaccine doses stratified by age. HCWs received either 2x BNT162b2 with (**a**) a short dose interval and a booster dose of BNT162b2 or mRNA-1273 (n=120) or (**b**) 2x BNT162b2 (n=62), (**c**) 2x mRNA-1273 (n=72), (**d**) ChAdOx1 + BNT162b2 (n=52), or (**e**) ChAdOx1 + mRNA-1273 (n=22) with a long dose interval. Antibody levels of all vaccinees are presented in panel **f**. SARS-CoV-2 S1-specific IgG antibody levels were measured 3 weeks after the first and the second vaccine dose (1D3wk, 2D3wk), and 3 months after the second dose (2D3mo) for all vaccinees. Also 3 weeks after the third booster dose (3D3wk) were measured for vaccinees' who had received three vaccine doses. Geometric mean antibody levels are shown as bars with geometric SDs. Cut-off value is indicated with a dashed line. Differences between age groups were analyzed using two-sided Kruskal-Wallis test with Dunn's multiple comparisons test. P-values <0.05 are considered statistically significant and p-values of <0.0001 are marked with ****. The other p-values from left to right in the panel **a** are *** 0.0003, * 0.0122, * 0.0149, ** 0.0041, ** 0.0055, * 0.0333 and ** 0.0011. P-values for panel **f** are *** 0.0008, ** 0.0026, * 0.0284, * 0.0263, ** 0.0099, and *** 0.0001.

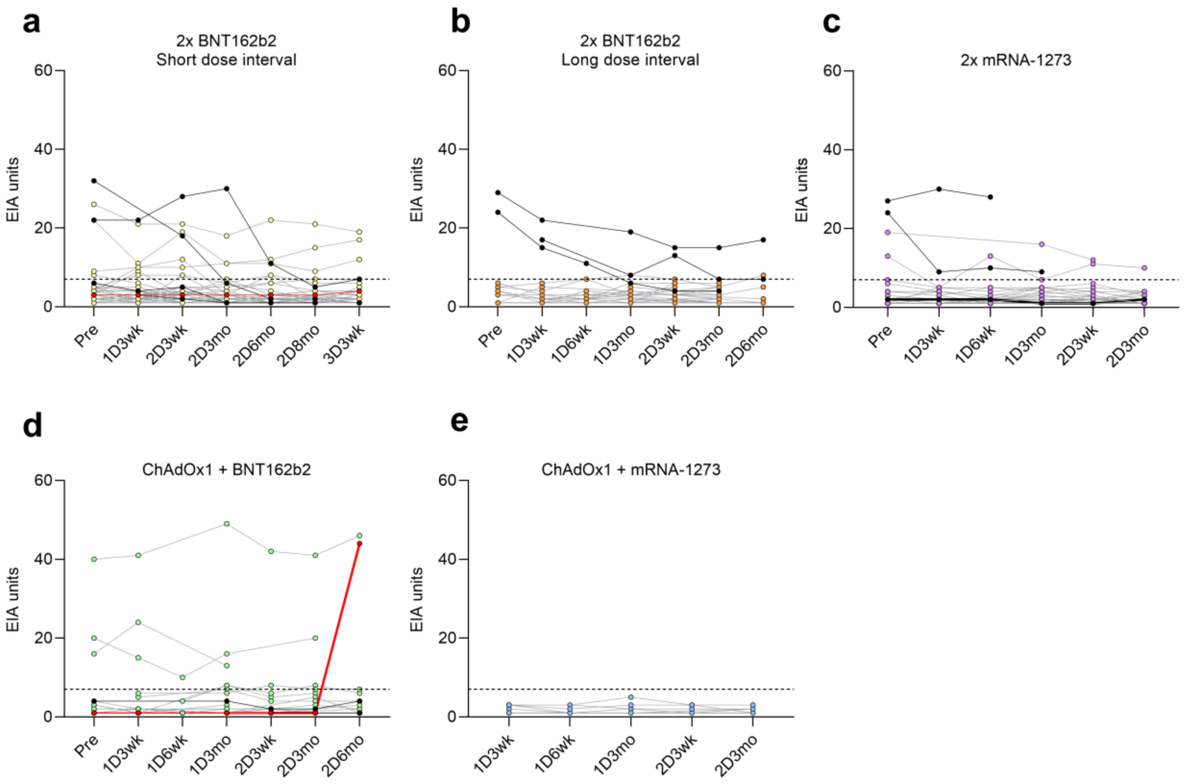

Supplementary Figure 3. SARS-CoV-2 N-specific antibody responses of HCWs during the follow-up. SARS-CoV-2 N-specific IgG antibody levels were measured in all vaccine combination groups: **a-b** 2x BNT162b2 (short and long dose interval), **c** 2x mRNA-1273 (long dose interval), **d** ChAdOx1 + BNT162b2 (long dose interval), and **e** ChAdOx1 + mRNA-1273 (long dose interval). Serum samples from HCWs in the short dose interval group were collected before the vaccination (Pre), three weeks after the first dose (1D3wk), three weeks (2D3wk), three months (2D3mo), six months (2D6mo), and eight months (2D8mo) after the second vaccine dose (2D). Serum samples from HCWs in the long dose interval groups were collected before vaccination (Pre), three weeks (1D3wk), six weeks (1D6wk) and twelve weeks (1D12wk) after the first vaccine dose (1D), and three weeks (2D3wk), three months (2D3mo), and six months (2D6mo), after the second vaccine dose (2D). Sample amounts are represented in Supplementary Table 1. Twelve HCWs with PCR confirmed SARS-CoV-2 infection prior to vaccinations are represented with black dots and two HCWs with a breakthrough infection (>20 EIA unit increase between sequential serum samples or a positive PCR test result) are represented with red dots. Sequential serum samples are connected with lines.

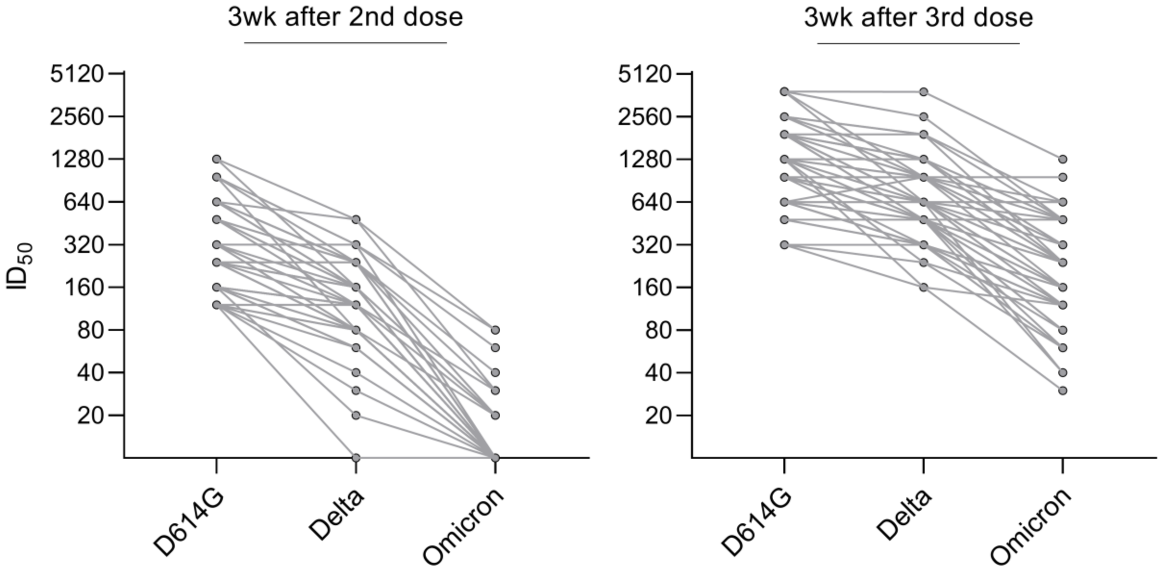

Supplementary Figure 4. Neutralizing antibodies against D614G, Delta and Omicron BA.1 after two doses of BNT162b2 vaccine and the third booster dose of BNT162b2 or mRNA-1273. Neutralizing antibody titers of HCWs (n=59) against D614G, Delta, and Omicron BA.1 variants three weeks after the second and third vaccine doses are compared. Each vaccinee's titers are connected with a line.

Supplementary Table 1. Amount of serum samples collected and the time before or after the first, second or third vaccine dose as indicated in the name of the timepoint.

		2x BNT162b2 short dose interval	2x BNT162b2 long dose interval	2x mRNA-1273 long dose interval	ChAdOx + BNT162b2 long dose interval	ChAdOx1 + mRNA-1273 long dose interval
Pre (days)	N	108/120	39/62	44/72	39/52	2/22
	mean	-12.0	-4.0	-5.8	-3.1	-2.5
	range	-129.0-7.0	-106.0-5.0	-167.0-0.0	-38.0-6.0	-5.0-0.0
1D3wk (weeks)	N	119/120	54/62	70/72	44/52	19/22
	mean	2.9	3.3	3.3	3.2	4.2
	range	2.3-4.0	2.9-4.3	2.7-5.0	2.4-4.6	3.0-5.3
1D6wk (weeks)	N	-	39/62	65/72	10/52	18/22
	mean	-	6.2	6.5	6.3	7.0
	range	-	5.4-7.6	5.6-8.0	5.4-7.4	5.9-8.0
1D3mo (months)	N	-	51/62	59/72	47/52	20/22
	mean	-	2.7	2.7	2.7	2.8
	range	-	2.1-3.5	2.5-2.9	2.4-2.9	2.6-2.8
2D3wk (weeks)	N	120/120	53/62	60/72	41/52	18/22
	mean	3.3	3.4	3.7	3.1	3.4
	range	2.4-5.7	1.9-4.9	2.6-5.0	2.0-4.9	2.3-3.9
2D3mo (months)	N	120/120	51/62	59/72	43/52	20/22
	mean	2.4	3.1	3.2	3.2	3.2
	range	2.0-3.5	1.9-4.0	2.5-3.9	2.9-3.8	3.0-3.7
2D6mo (months)	N	119/120	18/62	-	29/52	-
	mean	5.6	6.1	-	6.0	-
	range	4.4.-8.0	5.5-6.3	-	4.7-6.5	-
2D8mo (months)	N	109/120	-	-	-	-
	mean	8.1	-	-	-	-
	range	7.4-9.1	-	-	-	-
3D3wk (weeks)	N	120/120	-	-	-	-
	mean	3.6	-	-	-	-
	range	1.0-7.9	-	-	-	-
