# Supplementary material for: Comparative analysis of COVID-19 vaccine responses and third booster dose-induced neutralizing antibodies against Delta and Omicron variants
Source: Nat Commun. 2022 May 5;13:2476. doi: 10.1038/s41467-022-30162-5 (PMC9072399; doi:10.1038/s41467-022-30162-5)
Supplement: Supplementary file 3 — Reporting Summary [file 41467_2022_30162_MOESM3_ESM.pdf]

nature portfolio

Corresponding author(s): Milja Belik and Ilkka Julkunen

Last updated by author(s): April 1, 2022

Reporting Summary

Nature Portfolio wishes to improve the reproducibility of the work that we publish. This form provides structure for consistency and transparency in reporting. For further information on Nature Portfolio policies, see our Editorial Policies and the Editorial Policy Checklist.

Statistics

For all statistical analyses, confirm that the following items are present in the figure legend, table legend, main text, or Methods section.

n/a	Confirmed	
☐	☒	The exact sample size (*n*) for each experimental group/condition, given as a discrete number and unit of measurement
☐	☒	A statement on whether measurements were taken from distinct samples or whether the same sample was measured repeatedly
☐	☒	The statistical test(s) used AND whether they are one- or two-sided<br>*Only common tests should be described solely by name; describe more complex techniques in the Methods section.*
☐	☒	A description of all covariates tested
☐	☒	A description of any assumptions or corrections, such as tests of normality and adjustment for multiple comparisons
☐	☒	A full description of the statistical parameters including central tendency (e.g. means) or other basic estimates (e.g. regression coefficient) AND variation (e.g. standard deviation) or associated estimates of uncertainty (e.g. confidence intervals)
☐	☒	For null hypothesis testing, the test statistic (e.g. *F*, *t*, *r*) with confidence intervals, effect sizes, degrees of freedom and *P* value noted<br>*Give P values as exact values whenever suitable.*
☒	☐	For Bayesian analysis, information on the choice of priors and Markov chain Monte Carlo settings
☒	☐	For hierarchical and complex designs, identification of the appropriate level for tests and full reporting of outcomes
☒	☐	Estimates of effect sizes (e.g. Cohen's *d*, Pearson's *r*), indicating how they were calculated

Our web collection on statistics for biologists contains articles on many of the points above.

Software and code

Policy information about availability of computer code

Data collection	No software was used.
Data analysis	Excel 2016 (Microsoft 365), Prism v8 (GraphPad Software), and UCSF Chimera v1.15. (RBVI, University of California).

For manuscripts utilizing custom algorithms or software that are central to the research but not yet described in published literature, software must be made available to editors and reviewers. We strongly encourage code deposition in a community repository (e.g. GitHub). See the Nature Portfolio guidelines for submitting code & software for further information.

Data

Policy information about availability of data

All manuscripts must include a data availability statement. This statement should provide the following information, where applicable:

- Accession codes, unique identifiers, or web links for publicly available datasets
- A description of any restrictions on data availability
- For clinical datasets or third party data, please ensure that the statement adheres to our policy

All data are available upon request from the corresponding authors. Source data are provided with this paper. The SARS-CoV-2 sequences and protein data are available under accession codes PDB ID 6VXX, GenBank IDs MW717675.1 and MZ945494, and GISAID IDs EPI_ISL_412971, EPI_ISL_2557176, and EPI_ISL_8768822.2.

Field-specific reporting

Please select the one below that is the best fit for your research. If you are not sure, read the appropriate sections before making your selection.

[x] Life sciences [ ] Behavioural & social sciences [ ] Ecological, evolutionary & environmental sciences

For a reference copy of the document with all sections, see nature.com/documents/nr-reporting-summary-flat.pdf

Life sciences study design

All studies must disclose on these points even when the disclosure is negative.

Sample size	Due to the exploratory nature of the study, no sample-size calculation was performed. The study included 1015 serum samples from 208 individuals who completed a full two-dose vaccine regimen with a long 12-week (8.0–16.4 weeks) dose interval and 815 serum samples from 120 individuals who completed a full three-dose vaccine regimen with a short 3-week (2.6–4.0 weeks) dose interval between the first two doses. Neutralizing antibody responses were analyzed from 59 individuals from the short dose interval group.
Data exclusions	No data was excluded.
Replication	All serum samples were analyzed in duplicates in neutralization test and EIA. Internal quality controls included in each plate ensured the reproducibility.
Randomization	For the long interval group, all samples available at the time of the study were analyzed and no other randomization was performed. For the short interval group, we randomly selected the first 120 HCWs who received third vaccine dose from a larger cohort. The participation in the study did not modify the vaccination schedule of the participants and since the study is observational the covariates were not controlled.
Blinding	The study was not blinded for group allocation and principally all study participants were included into the study in an open label principle. The recruitment to the study was based on the time of vaccination.

Reporting for specific materials, systems and methods

We require information from authors about some types of materials, experimental systems and methods used in many studies. Here, indicate whether each material, system or method listed is relevant to your study. If you are not sure if a list item applies to your research, read the appropriate section before selecting a response.

Materials & experimental systems

n/a	Involved in the study	
[ ]	[x]	Antibodies
[ ]	[x]	Eukaryotic cell lines
[x]	[ ]	Palaeontology and archaeology
[x]	[ ]	Animals and other organisms
[ ]	[x]	Human research participants
[ ]	[x]	Clinical data
[x]	[ ]	Dual use research of concern

Methods

n/a	Involved in the study	
[x]	[ ]	ChIP-seq
[x]	[ ]	Flow cytometry
[x]	[ ]	MRI-based neuroimaging

Antibodies

Antibodies used	Polyclonal Rabbit anti-Human IgG conjugated to HRP (Dako A/S, P021402-2).
Validation	Validation of HRP conjugated antibody is available through manufacturers' website and they are tested by the manufacturer for specificity by immunoelectrophoresis and ELISA. Antibodies have also been tested in our previous publication (Jalkanen et al. NatCom 2021).

Eukaryotic cell lines

Policy information about cell lines

Cell line source(s)	VeroE6 cells (ATCC® CRL-1586) were obtained from the ATCC. VeroE6-TMPRSS2-H10 cells were obtained from Dr. Jussi Hepojoki (Rusanen et al. 2021, mBio).
Authentication	VeroE6 cells were authenticated by ATCC. VeroE6-TMPRSS2-H10 cells were not authenticated after transduction.
Mycoplasma contamination	All cell lines were tested negative for mycoplasma.

Commonly misidentified lines (See ICLAC register)	None

Human research participants

Policy information about studies involving human research participants

Population characteristics	Study participants were recruited among healthcare personnel of Turku University Hospital and Helsinki University Hospital prior to receiving vaccine as part of hospital occupational health care. The characteristics of participants were not pre-established. Study participants were 22-67 years old and 87% were female. Mean age for female were 44 years and for men 46 years.
Recruitment	The study participants were recruited among health care personnel at Turku University Hospital and Helsinki University Hospital. According to national vaccination priority principles health care workers involved in treating COVID-19 patients or COVID-19 diagnostics were vaccinated first and the vaccination programme started at the end of December 2020. Over 18 years old participants who received COVID-19 vaccination despite of the study were included without any exclusion criteria except Iv-immunoglobulin treatment. All vaccinees gave their written informed consent for the study. Age and gender distribution of participants represents well the study population and self-selection bias is not likely to impact the results.
Ethics oversight	The cohorts were approved by institutional review board of Southwest Finland health district and Helsinki-Uusimaa health district. At enrollment a written informed consent was collected from all participants.

Note that full information on the approval of the study protocol must also be provided in the manuscript.

Clinical data

Policy information about clinical studies

All manuscripts should comply with the ICMJEguidelines for publication of clinical research and a completedCONSORT checklist must be included with all submissions.

Clinical trial registration	The study is registered in EU Clinical Trials Register under EudraCT numbers 2021-004419-14 and 2021-004016-26.
Study protocol	Study protocols are available through EU CTR.
Data collection	Serum samples are collected 3-12 weeks and every three months after each vaccination dose at Turku University Hospital and Helsinki University Hospital starting in December 2020.
Outcomes	The study is an observational study on immune responses induced by three European Union licensed COVID-19 vaccines which are given to the participants as part of a national vaccination programme. The study thus does not include any predefined outcomes.
